# Studying the Structure and Viscosity of MnO-SiO_2_-CaO-Al_2_O_3_-MgO Slag System

**DOI:** 10.3390/ma17153789

**Published:** 2024-08-01

**Authors:** Shokouh Haghdani, Merete Tangstad, Kristian Etienne Einarsrud

**Affiliations:** Department of Materials Science and Engineering, Norwegian University of Science and Technology (NTNU), 7034 Trondheim, Norway; shokouhhaghdani@gmail.com (S.H.); merete.tangstad@ntnu.no (M.T.)

**Keywords:** manganese, silicate slags, structure, Raman spectroscopy, viscosity, structure–property relationship

## Abstract

The relationship between slag structure and viscosity is studied, employing Raman spectroscopy for the five-component slag system of MnO-SiO_2_-CaO-Al_2_O_3_-MgO and its subsystems. This study aims to investigate the influence of variations in slag composition on viscosity, which is crucial for optimizing industrial processes. Based on industrial slag compositions produced in a silicomanganese submerged arc furnace, 17 slags with a fixed content of MnO of 10 wt% are synthesized with varying contents of SiO_2_ of 33 to 65 wt%; CaO within the range of 14 to 40 wt%; and fixed contents of Al_2_O_3_ and MgO of 17 and 6 wt%, respectively. The slag compositions are divided into four groups, ranging from low basicity (0.38) to high basicity (0.80), with each group containing the four slag systems of MnO-SiO_2_-CaO, MnO-SiO_2_-CaO-Al_2_O_3_, MnO-SiO_2_-CaO-MgO, and MnO-SiO_2_-CaO-Al_2_O_3_-MgO, with fixed basicity. Additionally, a five-component composition with the lowest basicity of 0.28 is considered. Raman spectroscopy measurements are performed in the wavenumber range of 200 to 1200 ${\mathrm{cm}}^{-1}$ using a green source laser with a 532 nm wavelength. The high-wavenumber region of the Raman spectra (800 to 1200 ${\mathrm{cm}}^{-1}$) is deconvoluted to quantitatively investigate the effect of each oxide on the slag structure and the degree of polymerization (DOP) of the silicate network. Results indicate that measured NBO/T increases with increasing basicity, demonstrating a reduction in DOP of the silicate structure. This depolymerization effect is more pronounced in slags containing Al_2_O_3_ compared to those without it. In a group of slags with similar basicity, the substitution of SiO_2_ with Al_2_O_3_ leads to further depolymerization. In contrast, substituting CaO with MgO has little effect on the silicate structure in slags without Al_2_O_3_ but causes depolymerization in slags containing Al_2_O_3_. To study the relationship between structure and viscosity, viscosity data obtained from FactSage are used as reference values. The predictions of slag viscosity using the Raman-structure model and the NBO/T viscosity model are then compared to the FactSage results. The adjustable parameters of the Raman-structure model are re-determined using the FactSage data for the studied slag compositions. The NBO/T viscosity model employs both calculated NBO/T values from the slag compositions and measured NBO/T values from the deconvolution results. The findings of this study reveal good agreement between the predictions of the Raman-structure model and the FactSage viscosity data.

## 1. Introduction

Manganese ferroalloys are categorized as ferromanganese (FeMn) with varying carbon content (high, medium, or low) and silicomanganese (SiMn) [1]. High-carbon FeMn and SiMn are mostly produced through the carbothermic reduction of manganese ores with the addition of a carbon source in electric submerged arc furnaces. In manganese ferroalloys, the main element, manganese, is present withing the range of 60 to 80 wt%, while the content of silicon is typically less than 1 wt% for FeMn and between 16 and 30 wt% for SiMn [2]. In addition to manganese and silicon, iron and carbon are also present in manganese ferroalloys. The slag system produced in manganese ferroalloy processes is mainly composed of five oxides, namely MnO, SiO_2_, CaO, Al_2_O_3_, and MgO. Based on industrial slag compositions, the MnO content is between 15 and 40 wt% in FeMn slags, while for SiMn slags, the MnO and SiO_2_ contents are typically within 5 to 20 wt% and 40 wt%, respectively [1,2]. Knowledge of slag systems related to the primary production of FeMn or SiMn in submerged arc furnaces was reviewed extensively in a recent paper [2].

In high-temperature metallurgical processes such as the production of manganese ferroalloys, slag properties are of crucial importance because of their key roles in determining the performance of industrial operations [3,4]. Among these properties, viscosity has a significant impact on metal yield by affecting metal–slag separation efficiency [5] and the tapping process [6]. Slag viscosity is dependent on both temperature and slag composition; the viscosity decreases with increasing temperature. To better understand the relationship between viscosity and slag composition, studying the structure of slag is necessary [7,8,9,10,11,12]. Therefore, gaining insight into the atomic structure of molten slags is of fundamental importance in understanding and controlling metallurgical operations [9,10,11,12,13,14]. Among various spectroscopic techniques, the Raman spectroscopy method has been widely used to obtain valuable knowledge on the structural properties of numerous silicate melts and glasses [15,16,17,18,19,20,21,22,23]. The analysis of Raman data reveals the types of vibration species and their relative distribution through the examination of the peak shift and intensity of Raman spectra [24]. Silicate glasses are widely used as structural models for their corresponding melts due to the challenge of studying the structural properties of molten materials. It has been shown that silicate glasses and their molten counterparts possess similar structural properties when the glass is produced by rapidly cooling the molten slag [7,25].

Viscosity has been measured for slag systems containing MnO, including MnO-SiO_2_-Al_2_O_3_-CaO-MgO slag system [26,27,28,29] and its subsystems, for example, binary MnO-SiO_2_ [30,31], ternary systems of MnO-SiO_2_-CaO [30,32,33] and MnO-SiO_2_-Al_2_O_3_ [31], and quaternary systems of MnO-SiO_2_-CaO-MgO [34] and MnO-SiO_2_-CaO-Al_2_O_3_ [26,35]. Many Raman spectroscopy investigations of silicate melts and glasses have also reported data for slag systems containing MnO, such as the ternary systems of CaO-SiO_2_-MnO [36,37,38] and MnO-SiO_2_-Al_2_O_3_ [39], as well as the quaternary systems of CaO-SiO_2_-MnO-xCaF_2_ [x = 0.0 to 14.5 wt%] [38], TiO_2_-MnO (30 wt%)-SiO_2_-Al_2_O_3_ [40], MO-SiO_2_-MnO-yCaF_2_ [M(=Ca or Ba)O, y = 0 to 15 mol%] [41], and MnO-SiO_2_-Al_2_O_3_-zCe_2_O_3_ [z = 0.0 to 5.6 mol%] [39]. However, the structure of the five-component slag system in manganese ferroalloy production, namely MnO-SiO_2_-CaO-MgO-Al_2_O_3_, has not been investigated yet.

In this paper, slag structure is studied using the Raman spectroscopy technique for a group of 17 synthetic slags in the five-component slag system of MnO-SiO_2_-CaO-Al_2_O_3_-MgO and its subsystems, including MnO-SiO_2_-CaO, MnO-SiO_2_-CaO-Al_2_O_3_, and MnO-SiO_2_-CaO-MgO slag systems. In order to simulate industrial slags in silicomanganese production, fixed contents of MnO, Al_2_O_3_, and MgO oxides of 10, 17, and 6 wt%, respectively, are used, while the contents of SiO_2_ and CaO are varied from 33 to 65 wt% and 14 to 40 wt%, respectively. The slag compositions are divided into four groups, ranging from low basicity (0.38) to high basicity (0.80), with each group containing four slag systems with a fixed basicity. As in previous research [23,42,43,44], the Raman parameter (R) is calculated as the ratio of low-to-high-wavenumber vibrational bands within the range of 200 to 1200 ${\mathrm{cm}}^{-1}$ and is used in correlation with basicity (CaO + MgO/(SiO_2_ + Al_2_O_3_)), optical basicity [45], and non-bridging oxygen per tetrahedral cation (NBO/T) [9,45]. In this study, a strong correlation is found between R and the chemical parameters for slags containing Al_2_O_3_, where the values of R decrease as basicity, optical basicity, and NBO/T increase. However, for slags without Al_2_O_3_, R decreases slightly, particularly in correlation with NBO/T. The effects of basicity and various oxides on slag structure are studied by analyzing the Raman spectra in the high-wavenumber region (800 to 1200 ${\mathrm{cm}}^{-1}$) using deconvolution techniques. The results are discussed in detail. The predictions of viscosity models, such as the Raman-structure model and the NBO/T viscosity model, are compared to reference values obtained from FactSage 7.3 [46,47]. In the NBO/T viscosity model, both calculated NBO/T values from slag compositions and measured NBO/T values from deconvolution results are used to predict viscosity values. By comparing the predictions of viscosity models with FactSage results, the Raman-structure model predictions are found to be closer to the FactSage viscosity data. The results of this study can help in optimizing slag composition and improving the performance of metallurgical processes, such as silicomanganese production. Additionally, the findings can provide insights into the behavior of slags in various industrial settings.

This work is organized as follows. Section 2 describes sample preparation, characterization techniques, and Raman spectral analysis. In Section 3, the Raman spectroscopy results for the (10 wt%) MnO-SiO_2_-CaO-Al_2_O_3_-MgO slag system and its subsystems are presented, and the relationship between slag structure and viscosity is discussed. Finally, in Section 4, the key findings are summarized and conclusions are drawn.

## 2. Materials and Methods

### 2.1. Sample Preparation

This study investigated slag compositions containing 10 wt% MnO through Raman spectroscopy. The slags were selected based on the slag systems produced in a silicomanganese submerged arc furnace, composed of MnO, SiO_2_, CaO, Al_2_O_3_, and MgO. These compositions were designed to study the effects of SiO_2_, CaO, MgO, and Al_2_O_3_ on the polymerization of the silicate network, as SiO_2_ acts as a network-former oxide, while CaO, MgO, and MnO are network-breaker oxides. The effect of Al_2_O_3_ is more complex, as it can act as either an acidic or basic oxide depending on the amount of other oxides present in the slag system.

Table 1 presents the designed compositions with SiO_2_ contents ranging from 33 to 70 wt%; CaO contents between 14 and 40 wt%; and fixed contents of Al_2_O_3_ and MgO of 17 and 6 wt%, respectively. To study the effect of basicity on slag structure, the slag compositions were categorized into groups based on their basicity, which is calculated as the ratio of the sum of CaO and MgO (C + M) to the sum of SiO_2_ and Al_2_O_3_ (S + A), all in wt%. The S + A and C + M contents were fixed in each group to investigate the variation in slag structure by substituting 17 wt% of SiO_2_ with Al_2_O_3_ and 6 wt% of CaO with MgO. The S + A values were 70, 65, 60, 55, and 50 wt%, while C + M values were 20, 25, 30, 35, and 40 wt% for slag groups A, B, C, D, and E, respectively. The groups, classified based on their basicity, range from the lowest basicity of 0.28 in group A to the highest basicity of 0.80 in group E. Each group contains 4 different slag systems, including the MnO-SiO_2_-CaO ternary system, MnO-SiO_2_-CaO-Al_2_O_3_ and MnO-SiO_2_-CaO-MgO quaternary systems, and the MnO-SiO_2_-CaO-Al_2_O_3_-MgO five-oxide system, with a constant basicity.

The synthetic slags were produced in an induction furnace with a rating of 30 kW (custom-made at NTNU, Trondheim, Norway) using the analytical reagent oxides of MnO (99.00% purity, Alfa Aesar, Kandel, Germany), SiO_2_ (99.50% purity, Alfa Aesar, Kandel, Germany), CaO (99.95% purity, Alfa Aesar, Kandel, Germany), Al_2_O_3_ (99.00% purity, Alfa Aesar, Kandel, Germany), and MgO (99.00% purity, Alfa Aesar, Kandel, Germany). To ensure the accuracy of experiments, the CaO and MgO oxides were calcined at 1273 K for 2 h in a muffle furnace to decompose any hydroxides. The powders were then precisely weighted according to the designed compositions listed in Table 1 and mixed to achieve homogeneous mixtures. A high-purity molybdenum crucible was securely positioned within a high-purity graphite crucible using graphite felt. Then 40 g of powder mixtures was carefully placed inside the molybdenum crucible. The assembly process involved inserting a mica sheet and appropriately sized graphite felt into the copper coil of the induction furnace. Subsequently, the graphite crucible was carefully placed within the copper coil. During the experiments, temperature monitoring was conducted using a type-C thermocouple. The thermocouple was positioned inside a molybdenum thermowell tube with one end closed and fixed to the wall of the graphite crucible. Before initiating the heating process, the induction furnace was vacuumed to achieve a pressure of $10^{-2}$ mbar. It was then filled with Ar gas (99.999% purity) until the pressure reached 1000 mbar. This process was repeated twice. Finally, the furnace was filled with He gas (99.9996% purity) until the pressure reached 1040 mbar. To create the desired experimental conditions, the furnace was heated at a rate of approximately 30 to 50 K/min, reaching temperatures ranging from 1973 to 2043 K, depending on the composition. The temperature was maintained for 2 h to ensure the homogenization of the slag melts. After 2 h, the molten slags were quickly quenched into a copper mold inside the furnace, which was cooled by water. The quenching process was performed quickly to ensure that the temperature of the molten slags was still well above their melting points, resulting in a glassy state. The quenched slag masses varied depending on the composition, within a range of 10 to 30 g. A portion of the quenched samples (approximately 10 g) was then ground for further characterization using techniques described in Section 2.2.

### 2.2. Characterization Techniques

In this study, the prepared slag samples were analyzed using X-ray fluorescence (XRF), X-ray diffraction (XRD), and Raman spectroscopy techniques. The XRF method (Thermo Fisher Scientific, Degerfors, Sweden) was employed to measure the chemical compositions, with sample preparation conducted using the flux fusion method.

Phase analysis of the slag samples was performed by XRD using a Bruker D8 A25 DaVinciTM instrument (Bruker, Karlsruhe, Germany) equipped with CuK radiation (wavelength of 1.54 Å). The measurement range was defined from 10 to 80^∘^ with a step size of 0.03.

A WITec Alpha 300R laser confocal Raman spectrometer (WITec GmbH, Ulm, Germany) equipped with a green laser (532 nm) was used to obtain the Raman spectra of the slag samples. The spectra were recorded in the wavenumber range of 200 to 1200 ${\mathrm{cm}}^{-1}$ at room temperature to study the relationship between the slag structure and viscosity. The raw Raman data were processed using the cubic baseline B procedure to remove the baseline (or background) [42]. This method defines the baseline between two boundaries where there is no signal, and it passes through one or two invariant domains in the region of 600 to 850 ${\mathrm{cm}}^{-1}$ [42]. Baseline correction was carried out using Fityk software (https://fityk.nieto.pl/, accessed on 24 July 2024), which is open-source software for data analysis and nonlinear curve fitting [48].

### 2.3. Viscosity Calculations

In this study, FactSage 7.3 [46,47], a thermochemical software and database package developed by Thermfact/CRCT (Montreal, QC, Canada) and GTT-Technologies (Aachen, Germany), was employed for the calculation of liquidus temperature and slag viscosity. The FToxid and FactPS databases, which contain extensive thermodynamic data for various oxides and pure substances, respectively, were utilized for these calculations.

## 3. Results and Discussion

### 3.1. Characterization Results

The XRF analysis results for the chemical compositions of the 17 slag samples in wt%, along with their liquidus temperatures (T_*l*_ (K)) calculated by FactSage are presented in Table 2. The XRF compositions for slags A1 to A3 are unavailable due to the challenges in producing glassy slags using the current furnace and quenching process. The produced slags were partially crystalline despite several attempts, which can be attributed to their high viscosity. As a result, only slag A4 was considered as an example for the five-component slag system of MnO-SiO_2_-CaO-Al_2_O_3_-MgO with the highest SiO_2_ content of 53 wt% in group A. The compositions obtained from XRF analysis are normalized to 100% and utilized in the following sections. Note that the presence of Al_2_O_3_ and MgO in XRF analysis of samples without the planned addition of these oxides can result from contamination during sample preparation and analysis. Furthermore, even high-purity analytical reagent oxides, typically with a purity of around 99%, can contribute to the detection of Al_2_O_3_ and MgO in XRF analysis of samples without these oxides.

**Figure 1:**
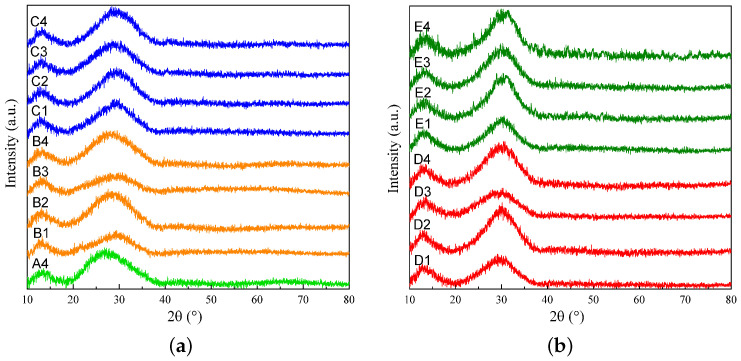
XRD results for (**a**) slags A4, B1 to B4, and C1 to C4 and (**b**) slags D1 to D4 and E1 to E4.

The results of the XRD analysis are presented in Figure 1; those for slags A4, B1 to B4, and C1 to C4 are shown in Figure 1a, and those for slags D1 to D4 and E1 to E4 are shown in Figure 1b. The XRD analysis shows that there are no crystalline peaks in the patterns, indicating that the quenched samples are in the glassy phase. The glassy state denotes the amorphous, non-crystalline structure that occurs when the molten slag is rapidly quenched. In contrast to crystalline materials, which possess a long-range ordered atomic structure, glassy materials are specified by a lack of periodic atomic arrangement, preserving the high-temperature structure of the slag [7,25].

#### Analysis of Raman Spectra

**Figure 2:**
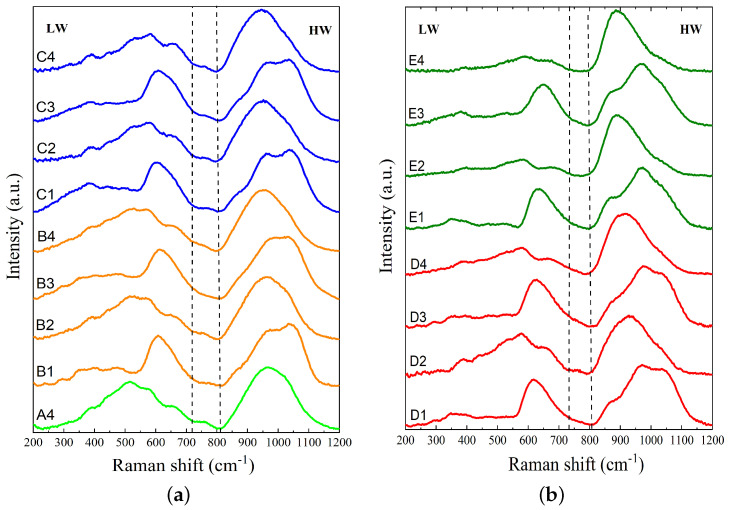
The normalized Raman spectra after baseline subtraction for (**a**) slags A4, B1 to B4, and C1 to C4 and (**b**) slags D1 to D4 and E1 to E4.

The normalized Raman spectra for slags A4, B1 to B4, and C1 to C4 are presented in Figure 2a, and the normalized Raman spectra for slags D1 to D4 and E1 to E4 are shown in Figure 2b. As seen in Figure 2, the Raman spectra of silicate glasses typically consist of two broad and asymmetric bands in the frequency range of 200 to 1200 ${\mathrm{cm}}^{-1}$. The first band, known as the low-wavenumber (LW) band, is located between ∼200 and ∼700 ${\mathrm{cm}}^{-1}$. As the degree of depolymerization of the silicate network increases due to higher basicity, this band experiences a shift towards higher wavenumbers and a decrease in intensity. The second band, also known as the high-wavenumber (HW) band, is located between ∼800 and 1200 ${\mathrm{cm}}^{-1}$ and has a center around 960 ${\mathrm{cm}}^{-1}$. With an increase in depolymerization of the silicate network, the HW band intensifies and shifts to lower wavenumbers. Additionally, a lower-intensity band can be detected at intermediate wavenumbers between 700 and 800 ${\mathrm{cm}}^{-1}$, with its peak located at around 750 ${\mathrm{cm}}^{-1}$. This is known as the medium-wavenumber (MW) band. The T–O^0^–T bending vibrations primarily produce the LW band, where T denotes Si or Al and O^0^ represents bridging oxygen. This region is typically assigned to the D1 (500 ${\mathrm{cm}}^{-1}$) and D2 (600 ${\mathrm{cm}}^{-1}$) bands, which are referred to as the breathing vibrations of four and three-membered rings of tetrahedral cations in the aluminosilicate network, respectively [49]. The MW band is generated by the stretching vibration of the T–O^0^ bond, where its intensity is related to the SiO_2_ content. In slags with Al_2_O_3_ and low basicity, the MW band is intensified [50]. The HW band is made by the stretching vibrations of T–O^−^ bonds, where O^−^ denotes non-bridging oxygen. The HW region results from the combination of aluminosilicate tetrahedral units known as Q^*n*^ species (n=0,1,2,3,and4), where Q represents a tetrahedron and *n* is the number of O^0^ per tetrahedron [51]. These units are denoted as monomer structure (Q^0^), dimer structure (Q^1^), chain structure (Q^2^), sheet structure (Q^3^), and a three-dimensional structure (Q^4^) [52]. The deconvolution of Raman spectra in the HW region allows for a quantitative study of these various silicate species. According to the literature, the bands related to Q^0^, Q^1^, Q^2^, Q^3^, and Q^4^ are centered at 850 to 880 ${\mathrm{cm}}^{-1}$, 900 to 920 ${\mathrm{cm}}^{-1}$, 950 to 1000 ${\mathrm{cm}}^{-1}$, 1050 to 1100 ${\mathrm{cm}}^{-1}$, and ∼1200 ${\mathrm{cm}}^{-1}$, respectively. However, the Q^4^ band is difficult to detect due to its low intensity. Hence, four Gaussian functions are allocated to the Q^*n*^ units (n=0,1,2,and3) to fit the Raman spectra in the range of 800 to 1200 ${\mathrm{cm}}^{-1}$. Deconvolution of Raman spectra was performed using OriginLab software 2018 [53].

### 3.2. Structure-Related Raman Parameter

Table 3 presents chemical composition parameters, including basicity ((B=(C+M)/(S+A))), optical basicity (OB), and non-bridging oxygen per tetrahedral cation (NBO/T) calculated using XRF compositions in mol%. The NBO/T is expressed as follows [9,45]:

(1) $$\frac{\mathrm{NBO}}{T}=\frac{2(X_{\mathrm{MnO}}+X_{\mathrm{CaO}}+X_{\mathrm{MgO}}-X_{{\mathrm{Al}}_{2}O_{3}})}{X_{{\mathrm{SiO}}_{2}}+2X_{{\mathrm{Al}}_{2}O_{3}}},$$

where $X_{i}$ denotes the mole fraction of oxide (i). Additionally, Raman parameters extracted from Raman spectroscopy measurements, as shown in Figure 2, are given in Table 3. The center of the Raman peaks in the low-wavenumber (LW) and high-wavenumber (HW) regions are represented by $C_{\mathrm{LW}}$ and $C_{\mathrm{HW}}$, respectively. The Raman parameter, (R), is calculated as the ratio of the intensity (I) of the two main bands of LW and HW, i.e., R = $I_{\mathrm{LW}}$/$I_{\mathrm{HW}}$ [43,44]. Previous research has shown that R values are inversely and non-linearly related to the chemical parameters, with R decreasing as B, OB, and NBO/T increase [23,43,44]. Thus, R serves as a representative parameter for the degree of polymerization of the silicate network in melts and glasses. In the presence of Al_2_O_3_, the intensity of the LW band decreases, while the intensity of the HW band increases, as observed in this study. A comparison between slags with and without Al_2_O_3_ shows that the addition of Al_2_O_3_ results in a reduction in the R values, indicating their role as network modifiers in slag systems. The shifts in $C_{\mathrm{LW}}$ and $C_{\mathrm{HW}}$ with varying slag compositions indicate changes in the polymerization or depolymerization of the silicate network in the slag structure. A shift in $C_{\mathrm{HW}}$ ($C_{\mathrm{LW}}$) towards the left (right), i.e., lower (higher) wavenumbers, indicates the depolymerization of the silicate network, while a shift towards the right (left), i.e., higher (lower) wavenumbers, indicates the polymerization of the silicate network in the slag structure.

**Figure 3:**
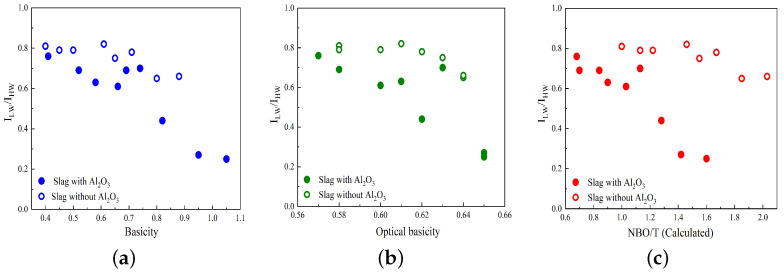
Variation in the Raman R parameter ($I_{\mathrm{LW}}$/$I_{\mathrm{HW}}$) versus (**a**) basicity, (**b**) optical basicity, and (**c**) NBO/T. The slags with and without Al_2_O_3_ are represented by solid circles and open circles, respectively.

Variations in the R parameter ($I_{\mathrm{LW}}$/$I_{\mathrm{HW}}$) against basicity, optical basicity, and calculated NBO/T are illustrated in Figure 3a–c. The slags containing and not containing Al_2_O_3_ are represented by solid circles and open circles, respectively. As seen in Figure 3, the R parameter decreases as basicity, optical basicity, and NBO/T increase for slags that contain Al_2_O_3_, which is consistent with previous studies [23,43,44]. For slags without Al_2_O_3_, such as the MnO-SiO_2_-CaO and MnO-SiO_2_-CaO-MgO systems, the R parameter decreases slightly with increasing basicity, optical basicity, and NBO/T.

### 3.3. Quantitative Analysis of Raman Spectra

**Figure 4:**
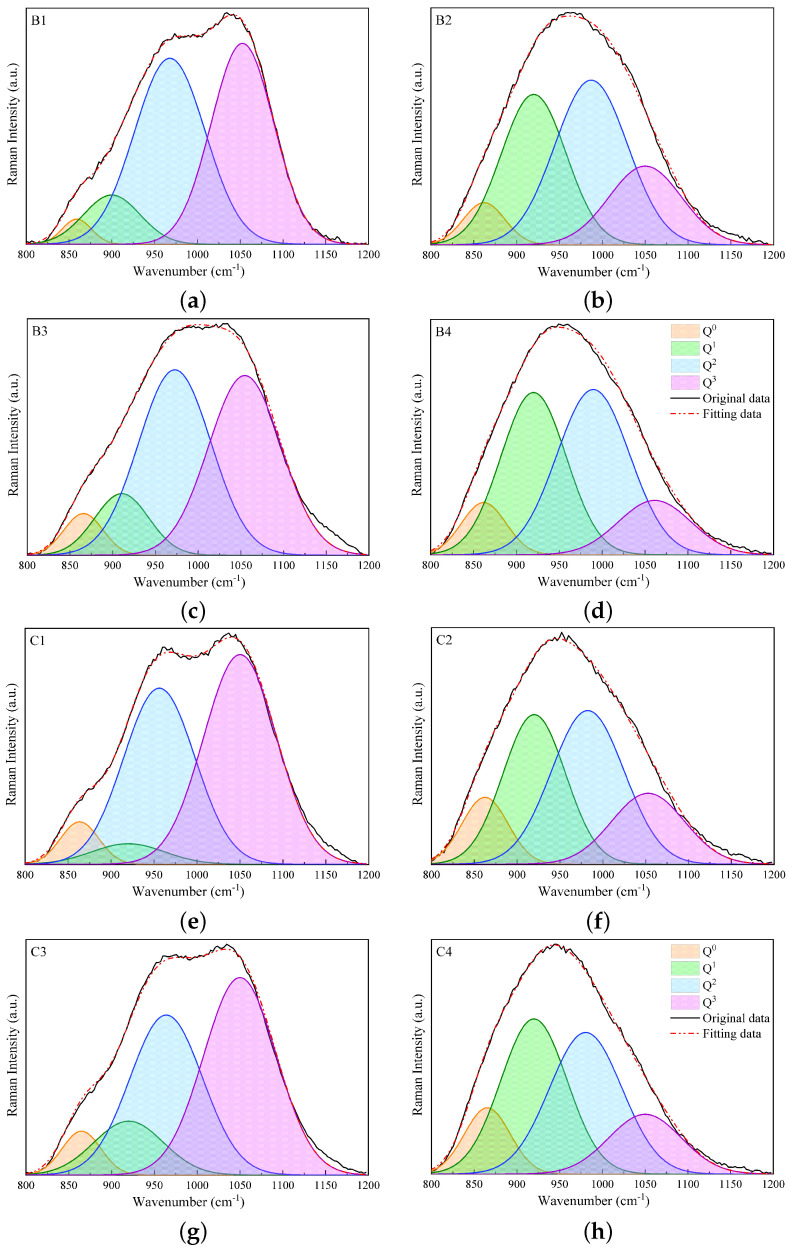
Fitting results of Raman spectra in the high-wavenumber region, i.e., 800–1200 ${\mathrm{cm}}^{-1}$, using Gaussian functions for slags B1 to B4 (**a**–**d**) and C1 to C4 (**e**–**h**).

**Figure 5:**
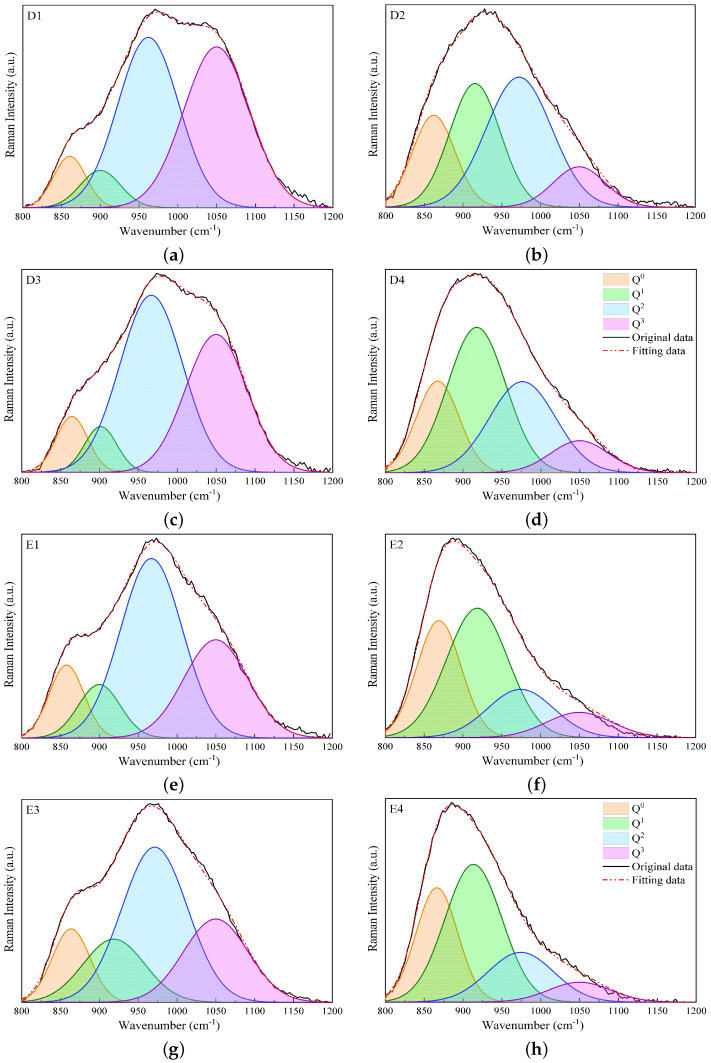
Fitting results of Raman spectra in the high-wavenumber region, i.e., 800–1200 ${\mathrm{cm}}^{-1}$, using Gaussian functions for slags D1 to D4 (**a**–**d**) and E1 to E4 (**e**–**h**).

In this section, the results of analyzing the different Q^*n*^ species (n=0,1,2,and3) obtained using the deconvolution of Raman spectra in the high-wavenumber range of 800 to 1200 ${\mathrm{cm}}^{-1}$ are presented. Figure 4 and Figure 5 display the fitting results for slags B1 to B4 and C1 to C4 and for slags D1 to D4 and E1 to E4, respectively. The bands of Q^2^ and Q^3^ species are predominant in slags without Al_2_O_3_ in groups B, C, and D, while the substitution of 17 wt% SiO_2_ by Al_2_O_3_ leads to a decline in Q^3^ and an increase in the Q^1^ and Q^2^ bands. In group E, the dominant band is Q^2^ for slags without Al_2_O_3_, while the predominant bands are Q^0^ and Q^1^ for slags containing Al_2_O_3_. In general, the study shows that the Raman spectra of slags containing SiO_2_ and slags containing both SiO_2_ and Al_2_O_3_ behave differently, while the Raman spectra of slags with CaO and slags with both CaO and MgO are relatively similar. Additionally, as the contents of SiO_2_ and Al_2_O_3_ are reduced, the Raman spectra in the high-wavenumber range shift to the left-hand side, and Q^*n*^ species with less bridging oxygen become predominant.

Table 4 presents the data extracted from the Raman spectral deconvolutions shown in Figure 4 and Figure 5, including the relative abundances (area; A_0_, A_1_, A_2_, and A_3_), and band centers (C_*n*_) of different Q^*n*^ species. For slags in groups B and C, the variations in A_*n*_ are similar, with A_2_ changing slightly and A_0_ having the lowest contribution. A_3_ and A_1_ are the predominant relative areas for slags without and with Al_2_O_3_, respectively, and their contributions switch when substituting Al_2_O_3_ with SiO_2_. For slags without Al_2_O_3_ in group D, namely slags D1 and D3, A_2_ and A_3_ are predominant, while A_0_ and A_1_ make fewer contributions. When Al_2_O_3_ is substituted for SiO_2_ in slags D2 and D4, A_3_ decreases, while A_0_ and A_1_ increase. For slags in group E, A_3_ makes fewer contributions, especially for slags with Al_2_O_3_ (slags E2 and E4), compared to the other groups. While A_2_ makes the main contribution for slags E1 and E3, A_0_ and A_1_ are predominant for slags E2 and E4.

The mole fractions of Q^*n*^ species, namely X_*n*_, are calculated using the A_*n*_ values presented in Table 4 according to the following equation [54]:

(2) $$X_{n}=\left(A_{n}/S_{n}\right)/\left(\sum_{n=0}^{3}A_{n}/S_{n}\right),$$

where S_*n*_ represents the Raman scattering coefficient for Q^*n*^ with values of $S_{0}$, $S_{1}$, $S_{2}$, and $S_{3}$ equal to 1, 0.514, 0.242, and 0.09, respectively [55,56].

The relationship between slag structure and viscosity can be quantitatively analyzed by the measured NBO/T value (${\left(\mathrm{NBO}/T\right)}_{\mathrm{mes}}$), which is obtained using X_*n*_ calculated according to Equation (2).

(3) $${\left(\mathrm{NBO}/T\right)}_{\mathrm{mes}}=\sum_{n=0}^{4}\left(4-n\right)\;X_{n}.$$

The ${\left(\mathrm{NBO}/T\right)}_{\mathrm{mes}}$ values are inversely proportional to the degree of polymerization (DOP) of the silicate network. This means that a higher NBO/T value corresponds to a lower DOP of the silicate network, typically resulting in a lower slag viscosity. The ${\left(\mathrm{NBO}/T\right)}_{\mathrm{mes}}$ values calculated by Equation (3) using deconvolution results are presented in Table 4.

**Figure 6:**
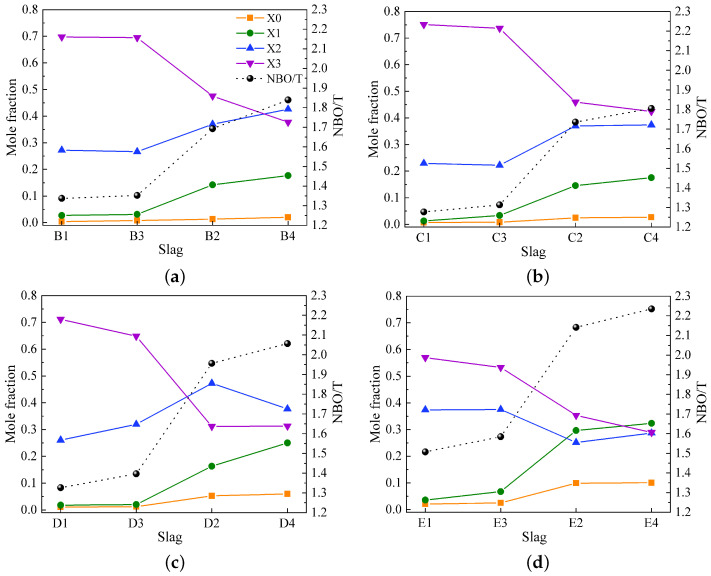
Variations in mole fractions for Q^*n*^ species (X_0_, X_1_, X_2_, and X_3_) and ${\left(\mathrm{NBO}/T\right)}_{\mathrm{mes}}$ values obtained from the deconvolution of Raman spectra for (**a**) slags in group B, (**b**) slags in group C, (**c**) slags in group D, and (**d**) slags in group E.

Figure 6a–d illustrate the variations in mole fractions for Q^*n*^ species (X_0_, X_1_, X_2_, and X_3_) and the ${\left(\mathrm{NBO}/T\right)}_{\mathrm{mes}}$ values for slags in groups B to E. The figures first present the variations in mole fractions and NBO/T for slags without Al_2_O_3_ (slags 1 and 3), where these variations are nearly identical. Subsequently, the variations for slags with Al_2_O_3_ (slags 2 and 4) are shown. Slags with similar basicity in each group show wide variations in both X_*n*_ and ${\left(\mathrm{NBO}/T\right)}_{\mathrm{mes}}$ values, with the variations being more pronounced between slags with and without Al_2_O_3_. In general, X_2_ and X_3_ have higher values, while X_0_ and X_1_ make fewer contributions to slag compositions in groups B to E, except for slags containing Al_2_O_3_ in group E, where X_1_ is more prominent. The substitution of 17 wt% SiO_2_ with Al_2_O_3_ leads to a decrease in X_3_, indicating the depolymerization of the silicate network. Substituting 6 wt% CaO with MgO has little effect on the silicate structure in slags without Al_2_O_3_. However, the substitution of 6 wt% CaO with MgO in slags containing Al_2_O_3_ causes the depolymerization of the silicate network, as seen by the reduction in X_3_ and the increase in X_1_.

**Figure 7:**
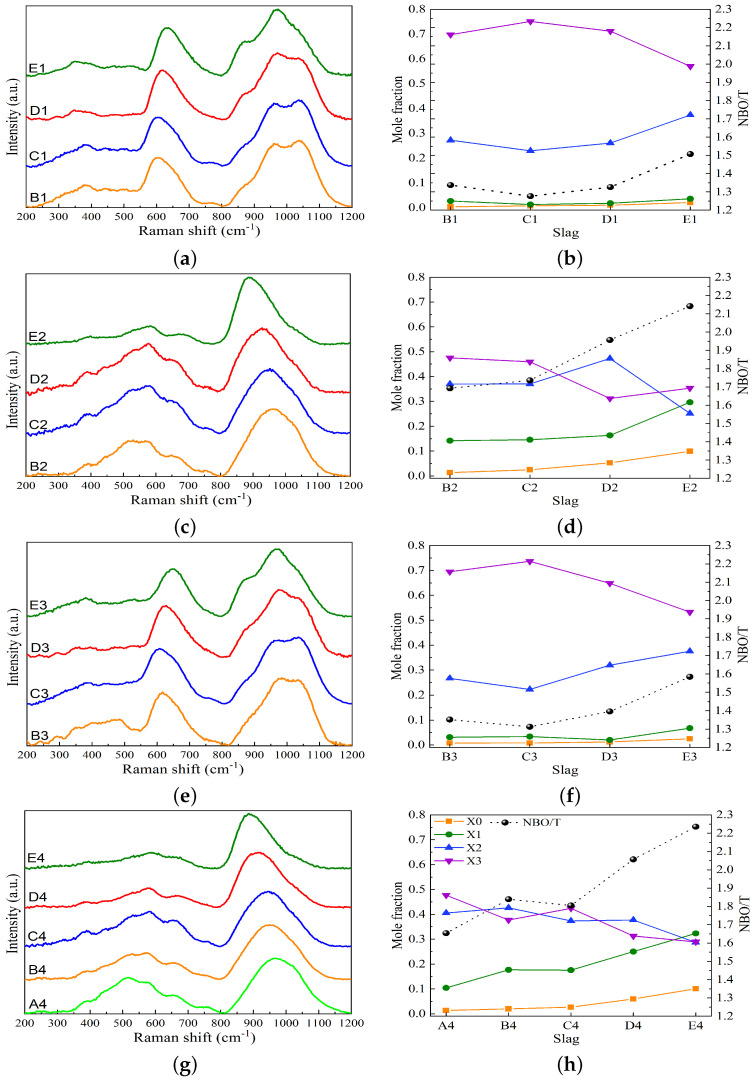
Raman spectra, the variations in X_*n*_ species, and the ${\left(\mathrm{NBO}/T\right)}_{\mathrm{mes}}$ values plotted against the basicity for the studied slag systems. (**a**,**b**) MnO-SiO_2_-CaO, (**c**,**d**) MnO-SiO_2_-CaO-Al_2_O_3_, (**e**,**f**) MnO-SiO_2_-CaO-MgO, and (**g**,**h**) MnO-SiO_2_-CaO-Al_2_O_3_-MgO. NBO/T values obtained using deconvolution of Raman spectra (dotted lines).

Figure 7 presents the Raman spectra as a function of wavenumbers, the variations in X_*n*_ species, and the ${\left(\mathrm{NBO}/T\right)}_{\mathrm{mes}}$ values for the studied slag systems. For the MnO-SiO_2_-CaO slag system, the Raman spectra are displayed in Figure 7a. In the high-wavenumber range of 800 to 1200 ${\mathrm{cm}}^{-1}$, the Raman spectra for slags B1 and C1 exhibit two peaks at ∼960 and ∼1050 ${\mathrm{cm}}^{-1}$, corresponding to Q^2^ and Q^3^ bands, respectively, and a shoulder at 870 ${\mathrm{cm}}^{-1}$ related to the vibrations of Q^0^ and Q^1^. As basicity increases, the intensity of the 1050 ${\mathrm{cm}}^{-1}$ band decreases and the intensity of the 870 ${\mathrm{cm}}^{-1}$ band increases, as seen in slag E1. Figure 7b shows that the mole fraction of X_3_ decreases and that of X_2_ increases to compensate for X_3_. This indicates that the silicate network is depolymerized by increasing the basicity at a fixed MnO content. These findings are consistent with Park’s research on the MnO-SiO_2_-CaO slag system [36]. For the MnO-SiO_2_-CaO-Al_2_O_3_ slag system, Figure 7c illustrates that the broad band in the high-wavenumber range shifts to the left-hand side as the basicity increases from slag B2 to slag E2. Therefore, the mole fraction of X_3_ decreases, while X_1_ and X_0_ increase, as seen in Figure 7d. The Raman spectral analysis of the MnO-SiO_2_-CaO-MgO slag system shown in Figure 7e,f is similar to that of the MnO-SiO_2_-CaO system. The results suggest that the substitution of 6 wt% CaO with MgO causes slight depolymerization in the silicate structure. For the MnO-SiO_2_-CaO-Al_2_O_3_-MgO slag system, the broad band in the high-wavenumber region shifts to the left-hand side with increasing basicity, as presented in Figure 7g. Figure 7f illustrates that the mole fractions of X_1_ and X_0_ continuously increase as the basicity increases. The substitution of 6 wt% CaO with MgO leads to depolymerization of the silicate network, as indicated by a comparison of Figure 7d,h. Figure 7 indicates that ${\left(\mathrm{NBO}/T\right)}_{\mathrm{mes}}$ increases with increasing basicity, demonstrating a reduction in the degree of polymerization (DOP) of the silicate structure. The effect of basicity on the depolymerization is more pronounced in slags containing Al_2_O_3_, as indicated by higher ${\left(\mathrm{NBO}/T\right)}_{\mathrm{mes}}$ values compared to slags without Al_2_O_3_.

**Figure 8:**
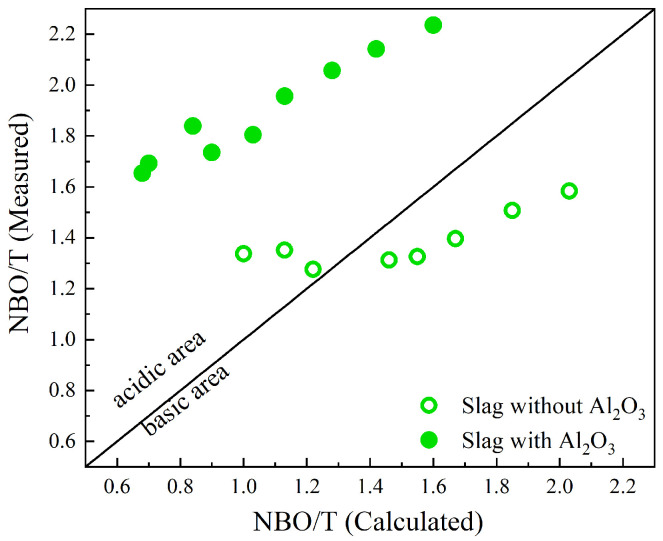
The relationship between calculated NBO/T values from slag compositions and measured NBO/T values from the deconvolution of Raman spectra, i.e., Equation (3). The slags with and without Al_2_O_3_ are represented by solid circles and open circles, respectively.

Figure 8 compares the calculated and measured NBO/T values, represented by ${\left(\mathrm{NBO}/T\right)}_{\mathrm{cal}}$ and ${\left(\mathrm{NBO}/T\right)}_{\mathrm{mes}}$, respectively. Slags with and without Al_2_O_3_ are represented by solid circles and open circles, respectively. The acidic (basic) region is identified where ${\left(\mathrm{NBO}/T\right)}_{\mathrm{mes}}$ values are higher (lower) than the ${\left(\mathrm{NBO}/T\right)}_{\mathrm{cal}}$ values [36]. This study expands Park’s research [36] by including slags containing Al_2_O_3_. Park utilized area fractions to determine measured NBO/T values for a CaO-SiO_2_-MnO slag system and found that the calculated NBO/T was significantly overestimated for values greater than 2.5 [36]. In this study, mole fractions calculated according to Equation (2) were used to calculate ${\left(\mathrm{NBO}/T\right)}_{\mathrm{mes}}$ values. For slags with Al_2_O_3_, the ${\left(\mathrm{NBO}/T\right)}_{\mathrm{cal}}$ values are significantly underestimated compared to the measured NBO/T, which may indicate an underestimation of the Al_2_O_3_ effect on the depolymerization of the silicate network. In comparison, for slags without Al_2_O_3_, the ${\left(\mathrm{NBO}/T\right)}_{\mathrm{cal}}$ values are relatively close to the measured NBO/T. However, for slags in groups D and E with calculated NBO/T values greater than 1.5, ${\left(\mathrm{NBO}/T\right)}_{\mathrm{cal}}$ is overestimated, while for slags in group B with calculated NBO/T values around 1, ${\left(\mathrm{NBO}/T\right)}_{\mathrm{cal}}$ is underestimated. As mentioned by Park, overestimation indicates that the silicate network is not as depolymerized as the calculated NBO/T suggests. Underestimation in the acidic region for slags without Al_2_O_3_ is the result of an underestimation of bridging oxygen, corresponding to Q^4^. This fully polymerized silicate unit was not determined in the deconvolution process due to the low intensity of the 1200 ${\mathrm{cm}}^{-1}$ bands in the Raman spectra. For slags containing Al_2_O_3_, the ${\left(\mathrm{NBO}/T\right)}_{\mathrm{cal}}$ values are significantly underestimated compared to the measured NBO/T, especially in slags with lower basicity. Note that the calculation of NBO/T assumes that all Al_2_O_3_ act as a network former in the presence of sufficient charge-balancing cations. However, research has revealed that a fraction of Al_2_O_3_ exists, even in peralkaline compositions, without being associated with a charge-balancing cations [57]. This fraction of Al_2_O_3_ acts as a network modifier, influencing the depolymerization of the silicate network and leading to larger measured NBO/T values compared to the calculated ones. In addition, the presence of aluminosilicate chains (Q^2^) or cation-deficient regions can contribute to the observed discrepancy between the measured and calculated NBO/T values in slags containing Al_2_O_3_ [50,58].

### 3.4. Relation between Structure and Viscosity

In this section, the correlation between slag viscosity and structure is presented using various parameters, such as the R parameter ($I_{\mathrm{LW}}$/$I_{\mathrm{HW}}$), calculated NBO/T from slag compositions (${\left(\mathrm{NBO}/T\right)}_{\mathrm{cal}}$), and measured NBO/T from the deconvolution of Raman spectra (${\left(\mathrm{NBO}/T\right)}_{\mathrm{mes}}$). Table 5 presents the viscosity values, denoted as η, for temperatures in the range of 1423 to 1873 K for all slag compositions, as obtained by FactSage [46,47]. These viscosity values from the FactSage software are used as reference data for comparing predicted viscosity values from different viscosity models. This choice is due to the unavailability of experimental viscosity values for all slag compositions from a single reference. Note that the Raman-structure model, the NBO/T model, and FactSage predict viscosity based on distinct slag structural parameters and/or viscosity–temperature equations.

**Figure 9:**
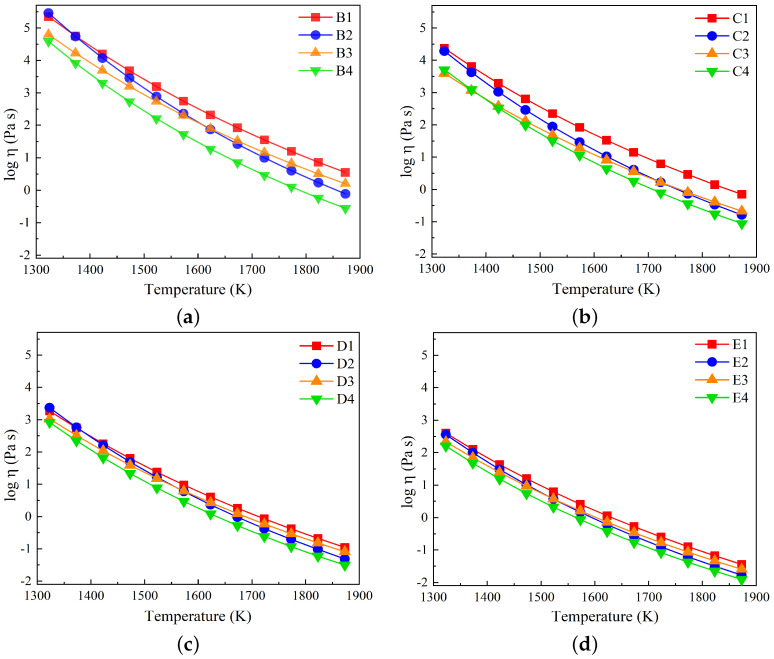
Variations in log η taken from FactSage against temperatures in the range of 1327 to 1873 K for (**a**) slags in group B, (**b**) slags in group C, (**c**) slags in group D, and (**d**) slags in group E.

For slags in groups B to E, the variations in log η are plotted as a function of temperature in Figure 9a–d. The viscosity decreases as the temperature increases for all slag compositions. Furthermore, slags in group B, characterized by the highest SiO_2_ content, have higher viscosity values compared to the corresponding slag compositions in other groups.

**Figure 10:**
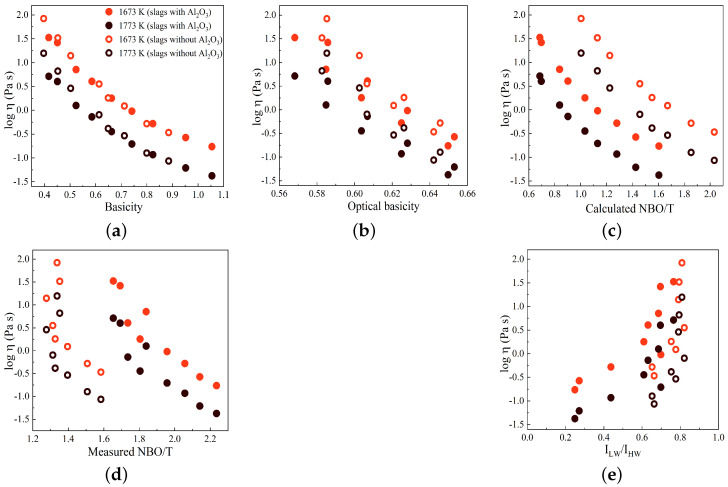
Variations in log η plotted against (**a**) basicity, (**b**) optical basicity, (**c**) calculated NBO/T, (**d**) measured NBO/T, and (**e**) Raman R parameter ($I_{\mathrm{LW}}$/$I_{\mathrm{HW}}$) at two temperatures of 1673 and 1773 K. Slags with and without Al_2_O_3_ are represented by solid and open circles, respectively.

In Figure 10a–e, the variations in log η are displayed against basicity, optical basicity, ${\left(\mathrm{NBO}/T\right)}_{\mathrm{cal}}$, ${\left(\mathrm{NBO}/T\right)}_{\mathrm{mes}}$, and the Raman R parameter ($I_{\mathrm{LW}}$/$I_{\mathrm{HW}}$), respectively, for two temperatures of 1673 and 1773 K. The slags with and without Al_2_O_3_ are indicated by solid circles and open circles, respectively. In general, as observed previously, the log η values decrease as the basicity, optical basicity, and NBO/T values increase. However, in contrast to this pattern, the log η values exhibit an increase with an increasing Raman R parameter, as depicted in Figure 10e. The R parameter serves as a representative measure of the polymerization degree within the silicate network, suggesting that an increased degree of polymerization corresponds to higher viscosity values. As shown in Figure 10c, the ${\left(\mathrm{NBO}/T\right)}_{\mathrm{cal}}$ values for slags containing Al_2_O_3_ are smaller compared to those for slags without Al_2_O_3_. However, the viscosity values for slags without Al_2_O_3_ are larger than the viscosity values for slags with Al_2_O_3_, contrary to the expected trend. This deviation can be attributed to the fact that the calculation of NBO/T assumes that all Al_2_O_3_ acts as a network former in the presence of sufficient charge-balancing cations. However, the results of this study indicate that a fraction of Al_2_O_3_ acts as a network modifier. This network modifier leads to the depolymerization of the silicate network, resulting in a decrease in viscosity, as expected. The variations in the R parameter and log η have similar patterns when plotted against basicity, optical basicity, and NBO/T parameters, particularly for slags containing Al_2_O_3_, as seen by comparing Figure 3 and Figure 10. This demonstrates the correlation between the Raman R parameter, structural chemical parameters, and viscosity, as reported in previous studies [23,43,44].

**Figure 11:**
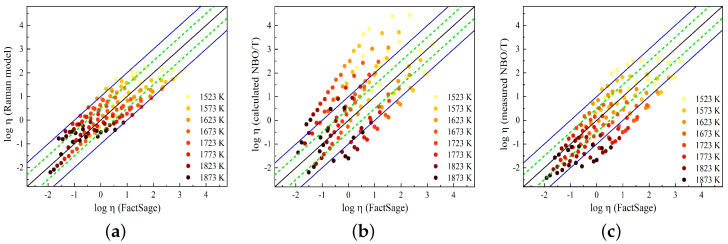
Comparison of viscosity values obtained from FactSage with viscosity values predicted by (**a**) the Raman-structure model, (**b**) the NBO/T model using ${\left(\mathrm{NBO}/T\right)}_{\mathrm{cal}}$ values calculated from XRF composition, and (**c**) the NBO/T model using ${\left(\mathrm{NBO}/T\right)}_{\mathrm{mes}}$ values obtained by Raman deconvolution analysis. The dashed lines represent a range of ±0.5 log units, while the solid lines represent a range of ±1 log units.

In Figure 11, the predicted viscosity values obtained using the Raman-structure model and the NBO/T model proposed by Giordano [59] are compared to the viscosity values taken from FactSage. In a previous study [23], the Raman-structure model was presented using the Arrhenius equation with four adjustable parameters for the SiO_2_-CaO-Al_2_O_3_ slag system. In this work, these parameters were re-determined using the viscosity data from FactSage (given in Table 5). By incorporating these new parameters, the viscosity model as a function of temperature and the Raman R parameter can be represented by the following equation:

(4) $$\log\;\eta =-12.44+1.06\;R+\frac{21350\;R^{0.095}}{T}.$$

Viscosity values at low temperatures, where FactSage calculations may be less precise, are included. These values could be corrected to account for partial solidification.

The NBO/T model, proposed by Giordano [59], employs the Tammann–Vogel–Fulcher (TVF) equation to express the temperature dependence of viscosity. This model is given by the following equation:

(5) $$\log_{10}\;\eta =a_{1}\;\ln(\mathrm{NBO}/T-a_{2})+a_{3}.$$

The adjustable parameters of $a_{1}$, $a_{2}$, and $a_{3}$ are temperature-dependent [59]. Figure 11a shows a comparison between the Raman-structure model results and FactSage, while in Figure 11b,c, the results of the viscosity model using ${\left(\mathrm{NBO}/T\right)}_{\mathrm{cal}}$ and ${\left(\mathrm{NBO}/T\right)}_{\mathrm{mes}}$, respectively, are compared to the FactSage results. Good agreement is observed between the Raman-structure model and FactSage viscosities, where the model predicts the FactSage viscosity within ±0.5 log units for most of the slags. The Giordano model overestimates the viscosity values for most of the slags when using the calculated NBO/T, while the model reproduces viscosity values closer to the FactSage values by using the measured NBO/T. In this study, the presence of Al_2_O_3_ in the slag compositions was found to result in a decrease in viscosity, indicating its network-modifying effect. While the calculated NBO/T considers to Al_2_O_3_ to act as a network-forming component, our measurements suggest that Al_2_O_3_ behaves as a network modifier. This discrepancy highlights the difference between viscosity values predicted based on ${\left(\mathrm{NBO}/T\right)}_{\mathrm{cal}}$ values and viscosity values obtained from ${\left(\mathrm{NBO}/T\right)}_{\mathrm{mes}}$ values, as displayed in Figure 11b,c. Figure 11 demonstrates that the Raman-structure model predicts the FactSage slag viscosity with good accuracy over a wide range of slag compositions and temperatures.

## 4. Conclusions

In this study, the relationship between slag structure and viscosity is investigated for a set of 17 synthetic slags that correspond to industrial slags in silicomanganese production. The slag viscosity is an important parameter when considering, for instance, metal–slag separation and tapping, thereby influencing metal yield. Raman spectroscopy is employed to analyze the slags, which include various combinations of MnO, SiO_2_, CaO, Al_2_O_3_, and MgO. The contents of SiO_2_ and CaO vary from 33 to 65 wt% and 14 to 40 wt%, respectively, while the contents of MnO, Al_2_O_3_, and MgO are fixed at 10, 17, and 6 wt%, respectively. The slags are classified into four groups based on their basicity from low basicity (0.38) to high basicity (0.80). The effects of basicity and the different oxides on slag structure are analyzed quantitatively using the results of the deconvolution of Raman spectra in the high-wavenumber region, i.e., 800 to 1200 ${\mathrm{cm}}^{-1}$.

The relationship between slag structure and viscosity is investigated by comparing the predicted viscosity values obtained from the Raman-structure model and the NBO/T viscosity model to the FactSage results. The adjustable parameters for the Raman-structure model are re-calculated for the studied slag set using FactSage data. Both calculated NBO/T values from slag compositions and measured NBO/T values from deconvolution results are used to predict viscosity in the NBO/T model. The results show good agreement between the Raman-structure model and FactSage. Among the results obtained using the NBO/T model, the predictions using measured NBO/T values are closest to the FactSage results. The findings of this study can be useful in optimizing slag composition and improving the efficiency of various metallurgical processes, such as silicomanganese production, as well as to gain a better understanding of the behavior of slags in different industrial systems.

## Figures and Tables

**Table 1:** Chemical compositions in weight percent of the MnO-SiO_2_-CaO-Al_2_O_3_-MgO slag system and its subsystems as designed in this study.

Slag	Designed Compositions (wt%)					Basicity
	${\mathrm{SiO}}_{2}$	CaO	MnO	${\mathrm{Al}}_{2}O_{3}$	MgO	
A1	70	20	10	0	0	0.28
A2	53	20	10	17	0	0.28
A3	70	14	10	0	6	0.28
A4	53	14	10	17	6	0.28
B1	65	25	10	0	0	0.38
B2	48	25	10	17	0	0.38
B3	65	19	10	0	6	0.38
B4	48	19	10	17	6	0.38
C1	60	30	10	0	0	0.50
C2	43	30	10	17	0	0.50
C3	60	24	10	0	6	0.50
C4	43	24	10	17	6	0.50
D1	55	35	10	0	0	0.63
D2	38	35	10	17	0	0.63
D3	55	29	10	0	6	0.63
D4	38	29	10	17	6	0.63
E1	50	40	10	0	0	0.80
E2	33	40	10	17	0	0.80
E3	50	34	10	0	6	0.80
E4	33	34	10	17	6	0.80

**Table 2:** Measured chemical compositions, in weight percent, of the MnO-SiO_2_-CaO-Al_2_O_3_-MgO slag system and its subsystems.

Slag	XRF Compositions (wt%)					Basicity	$T_{l}$ (K)
	${\mathrm{SiO}}_{2}$	CaO	MnO	${\mathrm{Al}}_{2}O_{3}$	MgO		
A4	51.4	13.5	10.5	17.0	7.5	0.31	1522
B1	65.6	24.2	9.1	0.8	0.2	0.37	1867
B2	48.6	24.2	9.3	17.5	0.4	0.37	1582
B3	64.8	18.2	9.5	0.8	6.7	0.38	1840
B4	47.1	18.0	10.2	17.5	7.2	0.39	1543
C1	61.6	28.7	8.8	0.6	0.2	0.46	1744
C2	43.6	28.9	9.3	17.7	0.5	0.48	1549
C3	58.9	24.6	9.0	0.8	6.8	0.52	1618
C4	42.8	23.0	9.4	17.6	7.0	0.50	1525
D1	55.9	33.8	9.3	0.8	0.2	0.60	1691
D2	39.1	33.6	9.0	17.7	0.5	0.60	1490
D3	55.5	27.8	9.1	0.8	6.8	0.61	1609
D4	38.4	27.9	9.3	17.4	6.9	0.62	1538
E1	51.8	38.6	8.7	0.7	0.3	0.74	1734
E2	33.9	38.6	8.8	17.9	0.6	0.76	1638
E3	50.7	32.6	8.9	0.7	6.9	0.77	1644
E4	33.1	32.8	9.2	17.7	7.2	0.79	1634

**Table 3:** The values of basicity (B), optical basicity (OB), and NBO/T, as well as the centers of the Raman peaks in LW and HW ranges ($C_{\mathrm{LW}}$ and $C_{\mathrm{HW}}$) in ${\mathrm{cm}}^{-1}$ and the R parameter (ratios of intensity heights (I) in LW and HW regions, $I_{\mathrm{LW}}$/$I_{\mathrm{HW}}$) for the studied slag systems.

Slag	B	OB	NBO/T	$C_{\mathrm{LW}}$	$C_{\mathrm{HW}}$	R
A4	0.41	0.57	0.68	517	968	0.76
B1	0.40	0.58	1.00	609	1034	0.81
B2	0.69	0.58	0.70	531	960	0.69
B3	0.45	0.58	1.13	614	1034	0.79
B4	0.52	0.58	0.84	567	949	0.69
C1	0.50	0.60	1.22	606	1037	0.79
C2	0.58	0.61	0.90	570	952	0.63
C3	0.61	0.61	1.46	609	1034	0.82
C4	0.66	0.60	1.03	584	944	0.61
D1	0.65	0.63	1.55	617	971	0.75
D2	0.74	0.63	1.13	578	928	0.70
D3	0.71	0.62	1.67	628	973	0.78
D4	0.82	0.62	1.28	584	914	0.44
E1	0.80	0.64	1.85	636	973	0.65
E2	0.95	0.65	1.42	584	887	0.27
E3	0.88	0.64	2.03	647	976	0.66
E4	1.05	0.65	1.60	589	885	0.25

**Table 4:** Deconvolution results of the Raman spectra, including the relative abundance (A_*n*_) in percent and the band center (C_*n*_) in ${\mathrm{cm}}^{-1}$, for each curve (Q^*n*^). The measured NBO/T values were calculated by Equation (3) using the deconvolution results.

Slag	Q^0^		Q^1^		Q^2^		Q^3^		${\left(\mathrm{NBO}/T\right)}_{\mathrm{mes}}$
	A_0_	C_0_	A_1_	C_1_	A_2_	C_2_	A_3_	C_3_	
A4	6.4	866	25.7	920	47.2	986	20.6	1057	1.65
B1	2.5	859	9.5	900	45.0	967	42.9	1052	1.34
B2	6.0	862	33.4	920	41.0	987	19.6	1050	1.69
B3	5.2	865	10.6	910	42.8	972	41.5	1054	1.35
B4	8.0	862	36.6	920	41.6	990	13.7	1061	1.84
C1	5.3	863	4.8	920	40.5	956	49.4	1050	1.28
C2	10.7	862	32.5	920	38.8	982	17.9	1053	1.73
C3	5.4	865	11.9	920	37.0	964	45.7	1050	1.31
C4	10.9	865	36.8	920	36.8	980	15.5	1050	1.80
D1	6.9	860	6.2	900	43.1	962	43.8	1050	1.33
D2	18.8	862	30.1	915	41.0	972	10.1	1050	1.96
D3	7.8	864	6.5	901	48.8	966	36.9	1050	1.40
D4	19.4	867	41.8	917	29.6	976	9.1	1050	2.06
E1	11.4	869	10.1	920	50.0	984	28.4	1050	1.51
E2	28.8	869	44.3	918	17.7	974	9.2	1050	2.14
E3	12.5	866	17.5	914	45.8	976	24.2	1050	1.58
E4	27.8	866	45.9	913	19.2	974	7.2	1050	2.23

**Table 5:** The viscosity values (η) for temperatures in the range of 1423 to 1873 K for all slags, taken from FactSage.

Slag	Temperature (K)									
	**1423**	1473	1523	1573	1623	1673	1723	1773	1823	1873
	**Viscosity (η) (Pas)**									
A4	64.68	34.97	19.83	11.73	7.21	4.59	3.01	2.04	1.41	1.00
B1	66.55	39.66	24.47	15.57	10.19	6.84	4.70	3.30	2.37	1.73
B2	58.61	31.69	17.95	10.61	6.51	4.13	2.71	1.82	1.26	0.89
B3	40.11	24.45	15.40	10.00	6.67	4.56	3.18	2.27	1.65	1.22
B4	27.08	15.34	9.08	5.59	3.56	2.34	1.59	1.10	0.79	0.57
C1	26.73	16.43	10.42	6.81	4.57	3.14	2.21	1.58	1.16	0.86
C2	20.49	11.71	6.98	4.32	2.77	1.83	1.25	0.87	0.62	0.45
C3	13.07	8.26	5.38	3.60	2.47	1.73	1.24	0.91	0.68	0.51
C4	12.41	7.34	4.52	2.88	1.90	1.29	0.90	0.64	0.47	0.35
D1	9.52	6.05	3.96	2.66	1.83	1.29	0.93	0.68	0.51	0.38
D2	9.07	5.42	3.37	2.17	1.44	0.98	0.69	0.49	0.36	0.27
D3	7.64	4.91	3.25	2.21	1.54	1.09	0.79	0.59	0.44	0.34
D4	6.17	3.80	2.42	1.60	1.08	0.76	0.54	0.39	0.29	0.22
E1	5.11	3.31	2.20	1.51	1.05	0.75	0.55	0.41	0.31	0.24
E2	4.40	2.74	1.77	1.17	0.80	0.56	0.41	0.30	0.22	0.17
E3	4.02	2.63	1.77	1.23	0.83	0.63	0.46	0.34	0.26	0.20
E4	3.28	2.09	1.38	0.94	0.65	0.47	0.34	0.25	0.19	0.15

## Data Availability

The raw data supporting the conclusions of this article will be made available by the authors on request.

## Abbreviations

The following abbreviations are used in this manuscript:

FeMn	Ferromanganese
SiMn	Silicomanganese
R	Raman parameter
NBO/T	Non-bridging oxygen per tetrahedral cation
XRF	X-ray fluorescence
XRD	X-ray diffraction
LW	Low-wavenumber
MW	Medium-wavenumber
HW	High-wavenumber
B	Basicity
OB	Optical basicity
I	Intensity
A	Relative abundance
C	Band left
X	Mole fraction
